# A comparative study of pre-trained language models for named entity recognition in clinical trial eligibility criteria from multiple corpora

**DOI:** 10.1186/s12911-022-01967-7

**Published:** 2022-09-06

**Authors:** Jianfu Li, Qiang Wei, Omid Ghiasvand, Miao Chen, Victor Lobanov, Chunhua Weng, Hua Xu

**Affiliations:** 1 School of Biomedical Informatics, The University of Texas Health Science Center at Houston, Houston, TX USA; 2 German National Library of Economics, Hamburg, Germany; 3 Covance by Labcorp, Princeton, USA; 4 Department of Biomedical Informatics, Columbia University, New York, USA

**Keywords:** Clinical trial, Eligibility criteria, Named entity recognition, Pre-trained language model

## Abstract

**Background:**

Clinical trial protocols are the foundation for advancing medical sciences, however, the extraction of accurate and meaningful information from the original clinical trials is very challenging due to the complex and unstructured texts of such documents. Named entity recognition (NER) is a fundamental and necessary step to process and standardize the unstructured text in clinical trials using Natural Language Processing (NLP) techniques.

**Methods:**

In this study we fine-tuned pre-trained language models to support the NER task on clinical trial eligibility criteria. We systematically investigated four pre-trained contextual embedding models for the biomedical domain (i.e., BioBERT, BlueBERT, PubMedBERT, and SciBERT) and two models for the open domains (BERT and SpanBERT), for NER tasks using three existing clinical trial eligibility criteria corpora. In addition, we also investigated the feasibility of data augmentation approaches and evaluated their performance.

**Results:**

Our evaluation results using tenfold cross-validation show that domain-specific transformer models achieved better performance than the general transformer models, with the best performance obtained by the PubMedBERT model (F1-scores of 0.715, 0.836, and 0.622 for the three corpora respectively). The data augmentation results show that it is feasible to leverage additional corpora to improve NER performance.

**Conclusions:**

Findings from this study not only demonstrate the importance of contextual embeddings trained from domain-specific corpora, but also shed lights on the benefits of leveraging multiple data sources for the challenging NER task in clinical trial eligibility criteria text.

## Introduction

### Background

Clinical trial protocols define important details about design and execution of clinical trials, which are the foundation for advancing medical sciences. An important section of clinical trials is the eligibility criteria (EC), which is often described in free text and not readily amenable for computer processing [1]. Formal representations developed in the past years have been used to optimize patient recruitment; but often require laborious manual effort to convert free text EC to structured representations [2, 3]. To address this challenge, natural language processing (NLP) techniques have also been investigated to process the EC text in clinical trials and convert them into standard representations in an efficient and effective manner [4, 5]. Named entity recognition (NER) is a fundamental and necessary step for extracting and standardizing EC using NLP. Recent deep learning approaches based on pre-trained language models such as Bidirectional Encoder Representations from Transformers (BERT) [6] have shown promising results in many NLP tasks including NER. Many transformer-based models using BERT and its variants have been studied for biomedical NER tasks, mainly for clinical notes in electronic health records (EHR) or articles in biomedical bibliographic databases. Few studies have applied BERT and its variants to NER tasks for clinical trial documents [5]. More specifically, there is no study that has systematically explored and compared performance of different BERT models on NER of EC in clinical trial documents.

In this study, we proposed to investigate different pre-trained language models (including both those trained from the general English domain and those specifically trained for the biomedical domain) for the NER tasks on EC of clinical trial documents. We systematically compared four biomedical domain-specific pre-trained contextual embedding models (named BioBERT [7], BlueBERT [8], PubMedBERT [9], and SciBERT [10]) and two general-domain models (named BERT and SpanBERT [11]), for extracting diverse types of clinically relevant entities from three annotated clinical trials corpora: (1) 470 in-house drug development study protocols annotated by Covance [5], (2) 230 Alzheimer’s disease (AD) clinical trial documents from ClinicalTrials.gov (named EliIE) [4], and (3) 1000 interventional, Phase IV clinical trials selected from ClinicalTrials.gov (named Chia) [12]. In addition, we investigated the feasibility of data augmentation approaches to leveraging different datasets to improve NER performance in EC.

### Related work

NER has been extensively studied and has shown its great use of supporting downstream applications in the medical domain, such as drug repurposing and clinical decision support [13, 14]. A lot of work has been focused on NER tasks for clinical reports, e.g., clinical concepts recognition, including rule-based, machine learning-based, and deep learning-based methods [15–21]. Many shared tasks have been organized and several annotated corpora of clinical notes have been created and made publicly available. For example, the well-known 2010 i2b2/VA Workshop on NLP Challenges for Clinical Records contained a task for concept extraction from clinical discharge summaries, the objective of which was to extract medical problems, treatments, and lab tests from patient reports [16]. Another example is the 2018 National NLP Clinical Challenges, which hosted shared tasks such as extraction of adverse drug events (ADEs) from narrative discharge summaries [17]. Recently, as the newly developed pre-trained language models including BERT and its variants achieved the state-of-the-art performance in a number of NLP tasks including NER, more and more studies have examined those pre-trained transformer-based models on NER tasks for clinical notes and reported superior performance [22, 23].

Clinical trial protocols, which provide detailed information about trial design and execution, are another type of important textual data in healthcare. In the past decade, researchers have worked on extracting and standardizing content of clinical trial documents (e.g., EC sections), with the goal to promote computerized applications during trial execution (e.g., automated criteria matching for trial recruitment). Different methods and tools have been developed for NER tasks that aim to extract key clinical concepts from EC and other sections of clinical trial protocols, including rule-based, machine learning-based, and hybrid methods [4, 24, 25]. In [4], an open-source information extraction tool called EliIE was developed, and it consists of four components: (1) entity and attribute recognition, (2) negation detection, (3) relation extraction, and (4) concept normalization and output structuring. EliIE used the conditional random field (CRF) algorithm for its NER task and achieved an overall F1 score of 0.786 on 7 types of entities. Zhang and Fushman [26] proposed rule-based strategies that extracted named entities using MetaMap and used them for classifying criteria. Yuan et al. [27] further developed a new natural language interface named Criteria2Query, which automatically transformed eligibility criteria to SQL queries for searching patients from clinical databases in OMOP Common Data Model. Like the EliIE tool, Criteria2Query also applied machine learning methods for NER and relation extraction tasks. More recently, Chen et al. [5] investigated deep learning models on NER from EC of clinical trials. In their study, BERT and BioBERT have been examined to extract entities from clinical trial protocols and they show improved performance, compared with traditional machine learning algorithms. Nevertheless, there is no comprehensive study that systematically investigates different contextual embeddings for NER in EC section of clinical trial documents. Recent state-of-the-art pre-trained language models that are developed for the biomedical domain (e.g., BlueBERT [8] and PubMedBERT [9]) have not been applied to clinical trial documents yet.

In addition, annotated corpora for NER in the EC section of clinical trial protocols have been developed in multiple studies, including (1) EliIE [4], which contains 230 annotated protocols of Alzheimer’s Disease (AD) clinical trial from ClinicalTrials.gov; (2) Covance [5], which contains 470 annotated drug development study protocols collected from in-house studies by Covance; and (3) Chia [12], which contains 1000 annotated protocols randomly selected from interventional Phase IV clinical trials registered in ClinicalTrials.gov. In addition to entities, both Covance and Chia also annotated modifiers to main clinical entities. Table 1 and 2 show some statistics of entities in the three corpora. Although such existing corpora provide great opportunities for method development and evaluation for NER in EC text, to the best of our knowledge, currently there is no study that has investigated NER approaches and systems across multiple clinical trial corpora.

**Table 1:** Basic information and statistics of entities in the three EC corpora for NER

Corpus	EliIE	Covance	Chia
Number of documents	230	470	1000
Source	Clinicaltrials.org	In-house by Covance	clinicaltrials.org
Disease Areas	Alzheimer’s disease only	All diseases	All diseases

**Table 2:** Main entities (entity types)—Count (number of occurrence) in the three EC corpora; numbers in the parentheses are nested occurrence for Chia corpus

EliIE		Covance		Chia	
Main entities	Count	Main entities	Count	Main entities	Count
Condition	4138	Condition	21,022	Condition	12,039 (127)
Drug	1465	Drug	13,671	Drug	3801 (24)
Qualifier	1715	Qualifier_Modifier	12,953	Qualifier	4157 (127)
Measurement	1029	Measurement	7732	Measurement	3305 (9)
Procedure_Device	652	Procedure	5635	Procedure	3595 (54)
Observation	1765	Observation	12,391	Observation	1216 (19)
Temporal_measurement	812	Temporal_constraint	11,326	Temporal	3580 (1066)
Anatomic_location	83	Anatomic_location	648	Negation	843 (0)
		Negation_Cue	1551	Device	386 (2)
		Event	4053	Multiplier	671 (8)
		Permission_Cue	2108	Person	1666 (2)
		Demographics	869	Value	4002 (60)
		Device	360	Visit	165 (1)
		Refractory_condition	662	Mood	616 (13)
		Investigational_product	559	Reference_point	934 (116)

The purpose of this study is twofold: (1) we want to systematically examine the performance of different state-of-the-art pre-trained language models (from both open domains and the biomedical domain) on NER for EC in clinical trial protocols; and (2) we plan to compare NER performance across multiple EC corpora and explore the feasibility of leveraging multiple data sources to improve NER performance in EC.

## Materials and methods

### Dataset

**Fig. 1:**
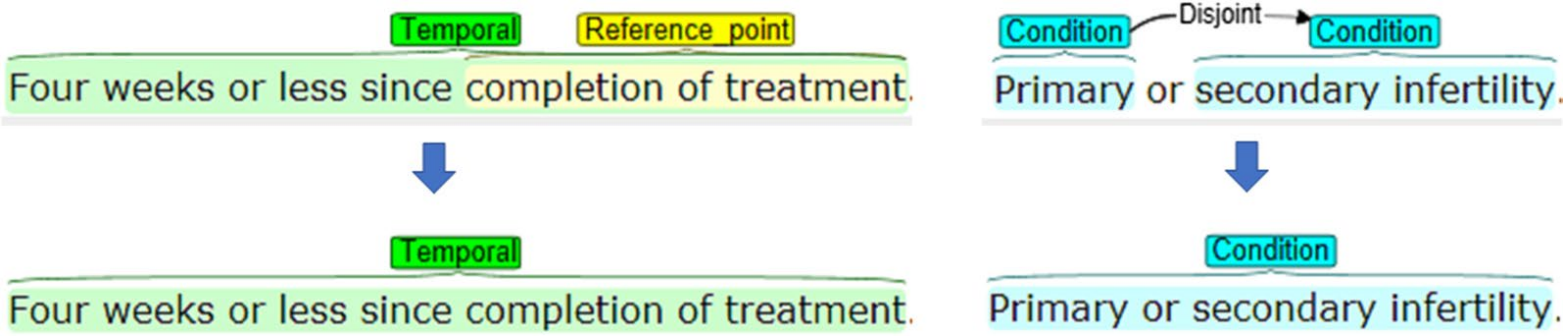
Examples of conversions of non-flat entities in the Chia corpus. Left: Nested entities; Right: Disjoint entities

In this study, we included all three corpora listed in Table 1: EliIE, Covance, and Chia. Among them, EliIE and Covance share similar annotation guidelines, although Covance contains more entity types than that in EliIE. The Chia corpus contains more fine-grained annotations of entity types and relations, e.g., including disjoint, nested, and overlapping entities. As such non-flat annotations require specific NER methods, we converted Chia annotations to continuous, non-overlapping entities only, to make them similar to EliIE and Covance annotations to ease the comparison. We applied two rules in this conversion: (1) for nested entities, we kept the outside entity only and removed the annotation of the nested one (Fig. 1-Left); and (2) we merged the disjoint entities to form a longer, continuous entity (Fig. 1-Right).

For Chia, there are two distinct datasets titled With Scopes and Without Scopes describing the inclusion or exclusion of Scope entities. The two datasets differs only in their utilization of Scope entity within the annotation model. We chose the Without Scopes dataset and combined the inclusion and exclusion together for each annotated EC file for our evaluation.

### Pre-trained language models

This study systematically investigated six state-of-the-art transformer-based language models: two from open domains: BERT and SpanBERT; and four for the biomedical domain: BioBERT, BlueBERT, PubMedBERT, and SciBERT.

BERT: A bidirectional deep transformer encoder model pre-trained on general domain corpora using masked language modeling (MLM) and next sentence prediction (NSP). The large model architecture has 24 transformer blocks with a hidden size of 1024 and 16 attention heads. The total number of parameters is 340 million. The model was trained on general English corpus from Wikipedia and BooksCorpus [28].

SpanBERT: A pre-trained transformer model extended BERT by: (1) masking contiguous random spans instead of random tokens, and (2) training the span boundary representations without relying on the individual token representations within it.

BioBERT: The first domain-specific BERT based model pre-trained on biomedical corpora. BioBERT was initialized with weights from BERT at first, then pretrained with additional corpus from large biomedical domain (PubMed abstracts and PMC full-text articles). BioBERT utilized WordPiece tokenization [29] to address the out-of-vocabulary issue so that any new words would be represented with subsequent subwords. It was shown to achieve better performance than the original BERT model on several biomedical NLP tasks like NER, relation extraction, and question answering.

BlueBERT: A pre-trained domain-specific transformer model by continual pretraining of BERT on biomedical and clinical corpora. Similar to BioBERT, BlueBERT was initialized with BERT firstly and then continue to pretrain the model using the large biomedical and clinical domain (PubMed abstracts and clinical notes MIMIC-III). The Biomedical Language Understanding Evaluation (BLUE) benchmark evaluated on five tasks with ten corpora shows that the BERT model pre-trained on PubMed abstracts and MIMIC-III clinical notes achieved better performance than most state-of-the-art models.

PubMedBERT: A pre-trained domain-specific transformer model by pretraining from scratch on a large biomedical domain. It generated the vocabulary and pre-trained from scratch to extend the uncased BERT Base model over a collection of PubMed abstracts and full PubMed Central articles.

SciBERT: A pre-trained domain-specific transformer model by pre-training from scratch on biomedicine and computer science domain. It generated the vocabulary and pre-trained from scratch to extend the cased BERT Base model over a random sample of 1.14 M papers from Semantic Scholar (18% papers from the computer science domain and 82% from PMC).

### NER using transformer models

**Fig. 2:**
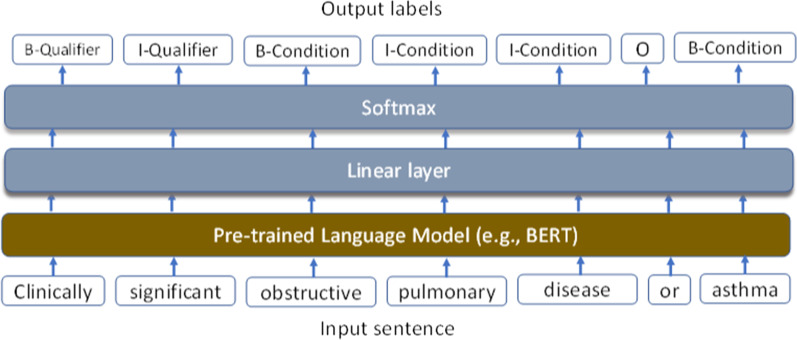
Architecture of the NER task using pre-trained transformer models

Figure 2 shows the architecture of the NER task using pre-trained transformer models. The NER task is formulated as a sequence labeling task, to assign a predefined B/I/O tag to each token of the sequence, where “B” represents the beginning of an entity, “I” represents tokens inside an entity, and “O” represents all other nonentity words. At first, the annotated sentences in each corpus were preprocessed and transformed into the “BIO” format (e.g., sentence boundary detection and initial tokenization) by CLAMP (Clinical Language Annotation, Modeling, and Processing toolkit) [30], then the input instances were processed by appending with a special token [CLS] at the beginning of the text. The processed inputs were tokenized based on the pre-trained language model’s vocabulary and then fed into the language model. Then the contextual representations of the tokenized processed input were generated. Finally, the NER task is done by using an additional linear classification layer on the contextual representations to predict token tags. To address the out-of-vocabulary (OOV) problem, the transformer models usually split original words into multiple pieces of sub-tokens, using a special tag “##” to be inserted in the front of the following sub-tokens.

All transformer models were downloaded from the HuggingFace website (https://huggingface.co/models). All NER models were trained using an NER package developed on the Transformers library implemented by the HuggingFace team [31] using PyTorch.

### Experiments and evaluation

For each corpus, a tenfold cross-validation (train/dev/test subsets with a ratio of 80%:10%:10%) was used to train and evaluate the performance of the NER models. Based on the state-of-the-art research in [35] and our previous experience, the following hyperparameters were used for all the models (Table 3).

**Table 3:** Hyperparameters used for all the transformer models

Hyperparameters	Value
training epochs	10
Learning rate	5.00E−05
Adam epsilon	1.00E−08
Training batch size	8
Maximum sequence length	256

We evaluated the performance of all the transformer-based NER models using both the strict and relaxed micro precision, recall, and F1-score [32], where strict means that an entity is correctly identified if both the boundary and entity type is same as those in gold standard, the relaxed means that an entity is correctly identified if its entity type is correct and its boundary overlaps with that in the gold annotations.

For the data augmentation experiment, we trained NER models by directly combining additional corpora (EliIE, Chia, and EliIE + Chia) with the training set of the Covance corpus and then evaluated their performance on the test set of the Covance corpus.

## Results

Table 4 shows the strict and relaxed micro P/R/F1 scores of six transformer-based models for NER in EC of trials on three corpora from Covance, EliIE, and Chia. Among all models, the PubMedBERT achieved the best performance on all three datasets, with strict and relaxed F1-scores of 0.715 (0.835), 0.832 (0.900), and 0.622 (0.744), respectively. To report the statistical significance of the differences among the results of the various experiments, the Wilcoxon rank sum tests [33], were also applied to compare the strict F1 metric of PubMedBERT with the other pre-trained models across the three corpora. Compared with the general domain pre-trained BERT model, the PubMedBERT improved the F1-scores by 1%, 2.9%, and 2.4% on Covance, EliIE and Chia corpora respectively. Different transformer models also showed consistent patterns for performance on the three corpora—all models achieved highest performance on the EliIE corpus and the lowest performance on the Chia corpus, with Covance in the middle. Moreover, the variations of the same model on different corpora were large (e.g., more than 20% in F1 score between EliIE and Chia), indicating the intrinsic differences between those annotated corpora in EC of trials.

**Table 4:** The strict and relaxed overall performance on the test sets of COVANCE, ELIIE, and CHIA corpora

Models	Covance			EliIE			Chia		
	P	R	F1	P	R	F1	P	R	F1
BERT	0.691 (0.810)	0.719 (0.849)	0.705 (0.829)	0.810 (0.877)	0.842 (0.917)	0.826 (0.896)	0.577 (0.701)	0.620 (0.761)	0.598 (0.730)
SpanBERT	0.692 (0.810)	0.718 (0.847)	0.705 (0.828)	0.813 (0.879)	0.843 (0.917)	0.828 (0.897)	0.593 (0.711)	0.628 (0.758)	0.610 (0.734)
BioBERT	0.694 (0.812)	0.722 (0.851)	0.708 (0.831)	0.810 (0.879)	0.837 (0.915)	0.823 (0.896)	0.589 (0.707)	0.632 (0.765)	0.609 (0.735)
BlueBERT	0.689 (0.807)	0.718 (0.848)	0.703 (0.827)	0.811 (0.880)	0.838 (0.917)	0.824 (0.898)	0.590 (0.702)	0.616 (0.737)	0.603 (0.719)
PubMedBERT	**0.704** **(0.820)**	**0.727** **(0.851)**	0.715* **(0.835)**	**0.817** **(0.881)**	**0.847** **(0.920)**	0.832* **(0.900)**	**0.606** **(0.724)**	**0.639** **(0.765)**	0.622* **(0.744)**
SciBERT	0.696 (0.813)	0.723 (0.850)	0.709 (0.831)	0.813 (0.883)	0.839 (0.915)	0.825 (0.899)	0.589 (0.709)	0.634 (0.768)	0.611 (0.737)

Bold values were calculated using the Wilcoxon rank sum test. The Wilcoxon rank sum test is a non-parametric test method that determines whether the means of strict F1 scores (Bold values) from the 10-fold experiments of the PubMedBERT model and each other model (BERT, SpanBERT, BioBERT, SciBERT) are statistically different from each other based on ranks rather than the original F1 scores of the experiments. The detailed definition of the Wilcoxon rank sum test can be found in the reference [33] as shown in the manuscript

Numbers in the parentheses are results based on relaxed criteria

*Indicates *p* < 0.05 when comparing to other pre-trained models

Table 5 shows the detailed results of the PubMedBERT model for each entity in the three corpora. Our results showed large differences in performance for different types of entities: F1-measures ranged from 0.429 to 0.830 for the Covance corpus, 0.507 to 0.881 for the EliIE corpus, and 0.015 to 0.808 for the Chia corpus.

**Table 5:** The strict performance of the PubMedBERT model for each main entity across the three corpora

Main entities	Covance			Main entities	EliIE			Main entities	Chia		
	P	R	F1		P	R	F1		P	R	F1
Condition	0.783	0.806	0.795	Condition	0.871	0.892	0.881	Condition	0.742	0.773	0.757
Drug	0.734	0.762	0.748	Drug	0.850	0.881	0.865	Drug	0.747	0.798	0.771
Qualifier_Modifier	0.597	0.599	0.598	Qualifier	0.780	0.814	0.796	Qualifier	0.444	0.486	0.462
Measurement	0.786	0.818	0.801	Measurement	0.863	0.871	0.866	Measurement	0.669	0.689	0.678
Procedure	0.651	0.674	0.662	Procedure_device	0.725	0.765	0.742	Procedure	0.574	0.630	0.600
Observation	0.651	0.679	0.664	Observation	0.754	0.792	0.771	Observation	0.278	0.260	0.267
Temporal_constraint	0.717	0.751	0.733	Temporal_measurement	0.807	0.829	0.815	Temporal	0.552	0.638	0.592
Anatomic_location	0.458	0.407	0.429	Anatomic_location	0.519	0.499	0.507	Negation	0.569	0.626	0.595
Negation_Cue	0.500	0.502	0.501					Device	0.528	0.515	0.520
Event	0.814	0.848	0.830					Multiplier	0.374	0.406	0.388
Permission_Cue	0.578	0.635	0.604					Person	0.795	0.824	0.808
Demographics	0.714	0.743	0.727					Value	0.727	0.745	0.735
Device	0.565	0.567	0.559					Visit	0.504	0.579	0.530
Refractory_condition	0.519	0.586	0.547					Mood	0.302	0.360	0.325
Investigational_product	0.657	0.630	0.641					Reference_point	0.398	0.524	0.453

Table 6 shows the results of the data augmentation experiments on common entities. When the EliIE corpus was added to the training set of the Covance corpus, it slightly improved the overall performance on the test set of Covance—F1 score was improved from 0.715 to 0.721. However, when Chia or Chia + EliIE was added to the training set of Covance, it dropped the overall F1 score on the test set of Covance.

**Table 6:** The strict performance for the common main entities of COVANCE with augment corpora using the PubMedBERT model

Main entities	Covance			Covance + EliIE			Covance + Chia			Covance + EliIE + Chia		
	P	R	F1	P	R	F1	P	R	F1	P	R	F1
Condition	0.783	0.806	0.795	0.784	0.808	0.796	0.765	0.801	0.783	0.767	0.799	0.782
Drug	0.734	0.762	0.748	0.734	0.761	0.747	0.731	0.754	0.742	0.727	0.756	0.741
Measurement	0.786	0.818	0.801	0.783	0.814	0.798	0.751	0.790	0.770	0.748	0.786	0.766
Observation	0.651	0.679	0.664	0.651	0.678	0.664	0.643	0.657	0.650	0.650	0.661	0.655
Procedure	0.651	0.674	0.662	0.652	0.660	0.656	0.636	0.665	0.650	0.632	0.663	0.647
Qualifier_Modifier	0.597	0.599	0.598	0.602	0.595	0.598	0.580	0.572	0.576	0.584	0.579	0.581
Temporal_constraint	0.717	0.751	0.733	0.720	0.751	0.735	0.707	0.750	0.728	0.707	0.748	0.727
Overall	0.704	0.727	0.715	0.712	0.731	0.721*	0.697	0.720	0.708	0.697	0.721	0.709

Bold values were calculated using the Wilcoxon rank sum test. The Wilcoxon rank sum test is a non-parametric test method that determines whether the means of strict F1 scores (Bold values) from the 10-fold experiments of the PubMedBERT model and each other model (BERT, SpanBERT, BioBERT, SciBERT) are statistically different from each other based on ranks rather than the original F1 scores of the experiments. The detailed definition of the Wilcoxon rank sum test can be found in the reference [33] as shown in the manuscript

*Indicates *p* < 0.05 when comparing to the original Covance corpus

Table 7 shows the computational time per epoch for all the models that trained on the three corpora using a single NVIDIA A100 GPU. Different models also showed consistent patterns for time complexity on the three corpora—all models spent longest time on the Covance corpus (with training data size 7.1 MB) and the shortest time on the EliIE corpus (with training data size 1.0 MB), with Chia in the middle (with training data size 4.0 MB).

**Table 7:** Computational time for training all the models on three corpora

Models	Training time (seconds per epoch)		
	Covance	EliIE	Chia
BERT	518.4	69.9	212.3
SpanBERT	520.3	70.5	212.3
BioBERT	343.4	30.9	92.6
BlueBERT	529.8	69.6	212.6
PubMedBERT	395.7	30.7	92.5
SciBERT	341.7	30.5	92.3

## Discussion

In this study, we systematically investigated general and domain-specific pre-trained language models for NER in EC text using three clinical trials corpora. Experimental evaluation shows that the PubMedBERT model achieved the best overall performance in all three corpora among six models. It achieved strict F1-scores of 0.715 and 0.832 on the Covance and EliIE corpora respectively, which were better than previously published results on these corpora (e.g., F1 of 0.708 for Covance in [5] and F1 of 0.786 on EliIE in [4]). These findings indicate that domain-specific language models are valuable for NER in EC and it worth further investigation.

BERT and SpanBERT were pre-trained using general corpora from English Wikipedia and BooksCorpus. Domain-specific models were built by either continuously fine-tuning on the top of BERT using biomedical corpora (e.g., BioBERT and BlueBERT) or training language models from scratch using biomedical corpora (e.g., PubMedBERT and SciBERT), thus providing more meaningful and representative word embeddings for downstream domain-specific tasks. As shown in Table 4, PubMedBERT and SciBERT also show slightly better performance than BioBERT and BlueBERT. One of the reasons could be that they have better vocabulary coverage on clinical trial documents, as they are trained from scratch using biomedical vocabularies. Table 8 shows the percentages of vocabulary coverage of BERT, PubMedBERT, and SciBERT on words from the three corpora of clinical trial protocols, which obviously indicates a smaller OOV problem for PubMedBERT. The reason that PubMedBERT outperformed SciBERT could be related to the training corpora—the SciBERT model was pre-trained from scratch using mixed domain corpora from both computer science and biomedicine. Nevertheless, the differences of performance between any domain-specific models are small.

**Table 8:** Percentages of vocabulary coverage of BERT, PubMedBERT, and SCIBERT in ELIIE, COVANCE, and CHIA

	EliIE (%)	Covance (%)	Chia (%)
BERT	47.5	28.1	34.3
PubMedBERT	63.2	44.4	53.4
SciBERT	54.8	34.1	41.9

A large performance variation was observed among three corpora (e.g., F1 scores of 0.715, 0.832, and 0.622 on Covance, EliIE, and Chia respectively, for the same PubMedBERT model), and patterns were consistent for all models (e.g., EliIE > Covance > Chia), which indicates the intrinsic differences among three annotated corpora, including (1) information models (e.g., types of entities and relations included); (2) annotation schemes and guidelines (e.g., whether to allow nested or disjoint entities); (3) sub-domains of samples (e.g., EliIE is from AD trials only); and (4) sample sizes. All models have better performance on the EliIE corpus probably due to that it contains trials from AD only and the types of entities are relatively simple. The low performance of Chia is probably mainly related to its complex and notable non-flat annotation schemes, as it stated that Chia was the first clinical trial corpus with considerable size annotated in a non-flat mode which supported annotations of nesting and disjoint entities [12]. When we applied rules to convert disjoint, nested or overlapping entities to continuous and non-overlapping entities in the preprocessing module, it may cause other issues such as reducing some types of entities while removing the inner nested entities or bringing certain noise while merging the disjointed entities, which would inevitably lower their performance. As the performance on Chia is not optimal, more advanced methods should be investigated to further improve NER systems to handle nested, disjoint, or overlapping entities in EC [34].

Our experiment that directly combined different corpora shows slight improvement when adding EliIE to the training set of Covance, the Wilcoxon rank sum tests show that the improvement is statistically significant with *p* < 0.05, therefore indicating it is worth investigating such data augmentation approaches for NER tasks in clinical trial documents. The reason that adding Chia to Covance did not improve the model performance is probably due to the differences of annotation schemes and guidelines between Covance and Chia. As stated in [5], the Covance corpus was constructed following a similar guideline as that of EliIE. Our next step is to investigate more sophisticated data augmentation algorithms, e.g.,, different domain adaptation methods [35–37].

There are limitations in this study. We mainly explored pre-trained language models on the NER tasks only. However, to support downstream applications, modifiers of clinical entities and standard codes of those entities should be identified as well. Therefore, our next step is to explore pre-trained language models on relation extraction tasks [15] in EC text. Furthermore, it is interesting to develop a robust mechanism to process the complex, non-flat annotations in Chia.

## Conclusion

In this study, we systematically compared BERT and its variants for NER in clinical trial eligibility criteria text and our results show that the PubMedBERT, which trained domain-specific language models from scratch using PubMed abstracts and full-text articles, achieved the best performance across multiple corpora, although variation among different models is small. However, large performance gaps were observed among different clinical trial corpora, calling for in-depth analysis of variations among different types of clinical trials, so that more generalizable approaches can be developed for all types of trial documents.

## Data Availability

The Chia dataset is publicly available on figshare at https://doi.org/10.6084/m9.figshare.11855817. To access the EliIE and Covance datasets, please contact Dr. Chunhua Weng and Dr. Miao Chen for further details and permission, respectively.
